# How a Brighter Outlook Has Been Brought about

**Published:** 1919-10-18

**Authors:** 


### THE FUTURE OF THE BLIND.

### I. How a Brighter Outlook Has Been Brought About.

The era of the blind man with his wheezy accordion and his pioneer dog, and of the patient, pathetic figure who read his embossed Bible in a monotonous mumble at the street corner is rapidly passing away. The whole question of the prevention of blindness and of the scientific care of its victims is being handled in a businesslike fashion, and is the object of increasing attention.

It is probable that the war has helped to bring about this better state of affairs. Without wishing for a moment to underrate the devoted endeavours of those workers who for many years have been endeavouring to awaken the public conscience, it may be said that the cause of the blind did not emerge from the slough of despond until one of the larger organisations commenced a vigorous campaign in the two years preceding the outbreak of war. To this effort we shall allude later; it appeared that for the blind the war had come at a moment when their hopes were brightest, and that the withdrawal of the recently awakened public attention would put back brighter days for many a year.

### How the War Helped the Blind.

The sufferer from the war who touched the sympathy of the public most of all was the soldier who had been blinded through his wounds. He was not unknown to history, of course; of the South African War a number of cases were the result. But during the recent great struggle so many men met with this unhappy fate that the need of a special institution for their training and welfare was quickly demanded. This was found in St. Dunstan's Hostel and its After-Care Department. Public interest has never ceased to be keen, and the blinded soldier has repaid the debt (if a debt it can be called) by helping his blinded brother-civilian. This is so because as knowledge of the disabilities of blindness came to the public their sympathy was extended to the cause of the blind generally.

In 1915 a Departmental Committee on the Welfare of the Blind was appointed by the Local Government Board, and the report issued in 1917 created an epoch in the cause. The main recommendation was that the general care and supervision of the blind should be a Ministry of Health. As a result of the report an Advisory Committee, very carefully chosen from educated and experienced blind men and those who had worked for the blind, was appointed by the Local Government Board. The first action taken by this Standing Committee was the preparation of a register of all blind persons—that is, of all persons who are too blind to perform work for which eyesight is essential. For this purpose the co-operation of all Public Health and Local Education Authorities, Institutions and Societies for the Blind, Nursing Associations, and Poor-Law Guardians was asked.

### The Labours of the Advisory Committee.

The first annual report of the Advisory Committee on the Welfare of the Blind has just appeared. One of their first steps was the arrangement of the registration with the Local Government Board of agencies for the blind, which had the result of the institution of a registry of every known agency which complies with three conditions, namely: (1) properly constituted committee of management, (2) properly audited accounts, and (3), in the case of workshops, payment of trade-union rates of pay in all branches of work where such rates exist. It is a matter for congratulation that insistence on these conditions has secured, in a number of cases, payment of such rates and the proper auditing of accounts. The report does not mention the result of the first condition; it is well known that many blind charities are branches of City Companies, and in these cases what would constitute a properly constituted committee would differ from the cases of ordinary charities. It is, however, very much a matter of definition. It is understood that the list will be subject to annual revision, and the Committee suggests greater stringency as to conditions in future years if the list is to be acceptable in all respects.

### Registration of the Individual Blind.

The numbers of the totally blind were ascertainable from the returns of the Census of 1911, but the figures did not include those who, though not totally blind, were too blind to perform work for which eyesight is generally considered necessary. A more comprehensive register was accordingly commenced, and a case form or questionnaire was drawn up, and copies were furnished to those bodies whose activities brought them in touch with the blind; at the same time wide notice was given in the Press as to the intention to compile the register. The substantial accuracy of the resulting register is not doubted by the Committee, but the accuracy of the figures, it must be remembered, depends wholly on the reliability to be placed on the particulars supplied from very various sources. In England and Wales 25,840 entries have been made. These are classified as to employment, and we make some quotations of interest. There are 24 blind clergymen, 81 domestic servants, 36 farmers, 439 hawkers, 85 beggars, 66 employed in massage, 464 musicians and singers, 212 newsvendors, 91 schoolmasters, etc., and 317 tuners (including musical instrument makers). The list was closed on January 1, 1919, but forms continue to be furnished from day to day, and it is expected that the total should come to some 30,000 in all.

### Employable and Unemployable.

A definition as to what constituted "unemployable" had to be arrived at, having regard to mental and physical defects, age, etc. After this had been done it was found that the unemployable group numbered 11,895, or 46 per cent. of the whole. Of this group 2,022 professed to live by means of private resources. It is considered that the "unemployable" class represents the real crux of the problem of dealing with the blind.

As to the employed group it may be said that in workshops there are employed 1,127 men and 455 women, while there are under training 396 men and 311 women. There are, of course, under training at St. Dunstan's and elsewhere persons not included in this total. The Census return of 1911 showed a total of 5,526 "occupied" blind persons, and the registry total is 6,391. It would seem, from a tabulated comparison between these two sources of information, that there has been an increase in the number of persons engaged in industrial occupations, and a decrease in those following non-industrial occupations, but it is thought that this is probably to be accounted for by the fact that the latter class are more likely to be of independent means and have not thought it necessary to furnish particulars.

Of the group of blind children there are 1,534 normally blind and 202 also mentally and physically defective, or deaf-blind, in schools and 362 apparently not at school,

**The Future of the Blind**—(*continued*).

40.6 per cent. of whom are noted as defective. In the experience of the members of the Committee further school accommodation is required, especially for blind children who are otherwise defective.

Industrial Conditions in Workshops.

In the large majority of cases it is impossible for the blind worker to earn a living wage at trade-union rates of pay, and this wage requires to be augmented. It is thought that the diversity of practice as to augmentation, by charity, of wages is neither equitable in itself nor acceptable to the blind workers, and that it is essential that a uniform system should be in operation throughout the country.

Attention is called to another difficulty—that of continuity of employment. During the war there was no lack of work in all branches of blind industry, but it is thought advisable to propose that in peace-time as much work as possible might be allotted to the workshops. It has been arranged for Government Departments to put as much work as circumstances admit at the disposal of blind workers.

The need of a uniform system of keeping the trading accounts of workshops is badly needed, and the Committee has suggested a form of account which might be adopted with advantage.

It is hoped, in a second article, to deal with the action taken by the Ministry of Health as a result of the Advisory Committee's report, and to allude to other current matters in respect of work amongst the blind.

(*To be continued.*)

